# Sex-specific patterns of avoidant and restrictive eating in inflammatory bowel disease: validation of the German version of the Nine Item Avoidant/Restrictive Food Intake disorder screen (NIAS)

**DOI:** 10.1186/s40337-026-01702-x

**Published:** 2026-07-03

**Authors:** Lea Pueschel, Stefanie Claire Fuhrberg, Leonie Kuhn, Josephine Conrad, Marina Florea, Navid Pueschel, Linda Schemann, Yilmaz Rohat Kala, Konstantina Atanasova, Laura-Louise Knoedler, Wolfgang Reindl, Eva Lina Wilke, Moritz Middelhoff, Anne Kerstin Thomann, Miriam Wiestler

**Affiliations:** 1 Department of Gastroenterology, Hepatology, Infectious Diseases and Endocrinology, Hannover Medical School, Hannover, Germany; 2 Department of Internal Medicine II, School of Medicine, TUM Klinikum Rechts der Isar, Technical University of Munich, Munich, Germany; 3 Department of Medicine II, Medical Faculty Mannheim, Heidelberg University, Mannheim, Germany

**Keywords:** ARFID, NIAS, Inflammatory bowel disease, Sex-differences, Eating disorders.

## Abstract

**Background:**

Avoidant/Restrictive Food Intake Disorder (ARFID) is a possible comorbidity in patients with Inflammatory Bowel Disease (IBD). Early detection and intervention are crucial to prevent malnutrition and related complications. However, ARFID often remains undiagnosed due to its subtle presentation. Methods: This multicenter study enrolled 235 patients with IBD at German tertiary referral centers between March and September 2025. The Nine Item Avoidant/Restrictive Food Intake disorder screen (NIAS), a screening tool for ARFID, was translated into German and administered at baseline and follow-up. The validity and reliability of the NIAS German were assessed via hypothesis testing and Cronbach’s α coefficient.

**Results:**

The NIAS German demonstrated excellent internal consistency (Cronbach’s α = 0.810) and test-retest reliability (*p* < 0.001). A significant correlation was found between the initial and follow-up NIAS scores for all items (*p* < 0.001). Furthermore, the intraclass correlation coefficient of 0.896 (95% CI: 0.846–0.931) confirmed the reliability of the German questionnaire version. Notably, women with IBD scored significantly higher on the NIAS and its subscales, indicating a greater likelihood and severity of ARFID symptoms, as screened by the NIAS (Md [IQR]: women = 13 [7–17]; men = 10 [6–15]; *p* = 0.007).

**Conclusions:**

The German version of the NIAS demonstrated good reliability and initial evidence supporting its use as a screening instrument in German-speaking men and women with IBD. The findings of this study highlight the importance of sex-specific assessment and suggest that IBD women may be at higher risk of developing ARFID. Early detection and targeted interventions are essential to prevent malnutrition and related complications in this vulnerable population.

## Abstract (plain language summary)

Living with an inflammatory bowel disease (IBD) can be challenging, and some patients may also experience a condition called Avoidant/Restrictive Food Intake Disorder (ARFID). ARFID can substantially impair dietary intake and may result in malnutrition and associated health complications. The present study sought to validate the translation of an ARFID screening tool (NIAS) into German. The study included 235 men and women with IBD and found that the NIAS German is a reliable instrument with potential utility for ARFID screening, pending further validation against diagnostic standards. The study also found that women with IBD were more likely to have ARFID than men. This observation is clinically relevant, as it suggests that women with IBD may require additional support and targeted interventions to address eating difficulties and mitigate the risk of malnutrition. Overall, the results advance efforts to identify and support individuals with ARFID in the context of IBD. The German NIAS provides healthcare professionals with a useful instrument to better assess patients’ needs and deliver more targeted interventions to optimize care.

## Background

Inflammatory bowel disease (IBD) and its two primary phenotypes Crohn’s disease (CD) and ulcerative colitis (UC) are characterized by recurrent and chronic inflammation of the gastrointestinal tract. Symptoms can include abdominal pain, nausea, and diarrhea, as well as complications such as fistulas, anemia, perforations, and stenoses [1], all of which may severely impact the quality of life (QoL) in patients with IBD. Current research findings demonstrate a significant influence of diet and nutrition on the etiopathogenesis of IBD, although its role in the course of the disease has not yet been conclusively clarified [2, 3]. Patient reports suggest subjective links between diet and disease activity [4, 5]. Indeed, there is a growing focus on evidence-based dietary therapies designed specifically for IBD populations, with ongoing research highlighting the importance of balancing nutritional status, symptom control, and QoL [6]. Certainly, for patients with IBD, nutrition is the central psychosocial concern [7–9] with food-related QoL in IBD being influenced by multiple factors including disease type, age, body weight, and sex, with the impact of diet varying; specifically, disease type affects men more, while older age and lower weight relate to better QoL in women, showing that dietary choices do not uniformly affect the QoL of patients with IBD, and that sex differences are an important factor [10].

Overall, while dietary measures can help control symptoms, food avoidance or selective eating driven by fear of symptom exacerbation can lead to nutritional deficiencies and complicate disease control [11]. Risk factors for the development of eating disorders are common among patients with IBD, and psychological stress and restrictive food intake are further associated with a reduced QoL [11, 12]. However, distinguishing between adaptive dietary adjustments and pathological patterns such as ARFID is clinically challenging, as these behaviors substantially overlap. ARFID is an eating disorder that imposes significant restrictions on an individuals’ dietary choices, can manifest at any age [13], and is not exclusive to IBD. Overall, the prevalence of eating disorders is often associated with body image disturbance, with a significantly higher incidence observed in women compared to men but in comparison, ARFID, which involves restrictive eating without concerns about body weight, appears to affect both sexes equally [14–16]. The condition is characterized by three distinct eating patterns: picky eating, a low appetite, or limited eating interest, in conjunction with a fear of negative gastrointestinal (GI) symptoms. It is further associated with a range of physical and psychosocial consequences, including, but not limited to, nutrient deficiency, weight loss, and psychosocial withdrawal [13, 15].

The “Nine Item Avoidant/Restrictive Food Intake disorder screen” (NIAS) [17] was developed to briefly screen for ARFID and has been translated into French [18], Spanish [19], and Turkish [20]. However, no validated German-language version currently exists. Nevertheless, studies show that 10% to 54% of patients with IBD meet the NIAS criteria for ARFID [21], underscoring the need for adequate diagnostic tools. For a comprehensive dietary management in German-speaking patients with IBD that integrates clinical, behavioral, and psychosocial dimensions to optimize patient outcomes and support sustained nutritional health, while also accounting for patients’ sex and gender, the German version of the NIAS will be a valuable additional tool. Therefore, the present study sought to meticulously translate the NIAS into German, and to validate this translation. In addition, patients with IBD constitute a distinct group, with sex- and gender having been identified in the past as salient influencing and differentiating factors overall [22] and in relation to diet and nutrition [10] with men for example exhibiting a tendency for a more adaptive eating behavior than women with IBD [23]. While research into ARFID is still ongoing, the distribution appears to be somewhat more balanced in terms of sex and gender than other eating disorders [15, 24, 25]. However, there is a paucity of data on sex- and gender differences of ARFID in patients with IBD. This study therefore seeks to further analyze the extent to which men and women with IBD differ in the context of ARFID symptoms, as screened by the NIAS.

## Materials and methods

### Ethical considerations

Data were obtained in the context of a multicentric study investigating nutritional aspects of patients with IBD. This multicentric study has been approved by the lead ethics committee of the Medical Faculty Mannheim on May 14, 2024. The study design is in accordance with the Declaration of Helsinki (2024). In addition, the study was pre-registered at aspredicted.org on January 30, 2025 (registration number: 210,298).

### Participants and setting

In the period between March and September 2025, a total of 253 individuals with IBD were screened in this multicenter study at three tertiary referral centers. Prior to screening, written informed consent was mandatory for each participant. The study inclusion criteria were met by individuals who had been diagnosed with IBD for a minimum period of three months and were between the ages of 18 and 80 years. Exclusion criteria encompassed symptomatic stenosis, a diagnosed eating disorder, confirmed intolerance to lactose, fructose, or gluten, known food allergies, and a planned change in diet regime.

### Data sources/measurements

A thorough data collection approach was used, involving a survey that gathered information on participants’ demographics such as sex and gender identity, age, marital status, and employment. Participants provided their height and weight, allowing for calculation of body mass index (BMI). Participants also completed a battery of validated questionnaires including the Visceral Sensitivity Index (VSI) [26, 27], which is designed to measure anxiety specifically related to gastrointestinal symptoms and has been successfully used in patients with IBD [28]. In addition, the FACIT Fatigue [29–31] scale was used to assess the severity and impact of fatigue on daily functioning, which is a common symptom in chronic illnesses including IBD. The Hospital Anxiety and Depression Scale (HADS) [32] was used to assess anxiety and depression. Further measures encompassed the Short Health Scale (SHS) [33–35], utilized for the assessment of IBD health-related QoL; the Screening Questionnaire for Highly Processed Food Consumption (sQ-HPF) [36, 37], designed for the evaluation of participants’ intake of processed foods; and the Mediterranean Diet Adherence Screener (MEDAS) [38, 39]. In addition, IBD food-related QoL was assessed via the FR-QoL-29 questionnaire [40, 41]. All questionnaires were provided in the German language. The survey also included questions about participants’ history of IBD including treatments, surgeries, and other health conditions. The classification of diseases followed the Montreal system for Crohn’s disease, and anatomical patterns for ulcerative colitis [42]. The assessment of disease activity and remission status was conducted utilizing specific indices: the Harvey–Bradshaw Index (HBI) [43] for Crohn’s disease and the partial Mayo score (PMS) for ulcerative colitis [44].

### Nine Item Avoidant/Restrictive Food Intake disorder screen (NIAS)

Permission to translate the original English-language questionnaire [17] into German was obtained a priori. As the NIAS originated in the United States, a forward-backward translation protocol, optimized for cross-cultural adaptation, was systematically applied [45, 46]. Two bilingual researchers independently translated the NIAS from English into German, ensuring both linguistic fidelity and contextual relevance. These preliminary translations were then subjected to a team-based review process before being translated back into English by an independent native speaker without prior knowledge of the original NIAS. Recognizing the complexity of the NIAS which is designed to evaluate pathological patterns in dietary behavior, the research team comprised of members with domain-specific knowledge in gastroenterology, clinical nutrition, dietetics, psychology, linguistics, and data analysis, thus ensuring the integrity of the German translation and appropriateness for the target population. The resolution of discrepancies between the versions was facilitated by inter-team deliberations. Subsequently, a preliminary field test was undertaken with ten student volunteers, intending to assess the comprehensibility of the German version. Participant feedback was systematically gathered through in-depth interviews with the insights gained informing subsequent linguistic and structural refinements. The final version of the translated NIAS includes nine items as does the original English version. The present study also aims to explore the three distinct subscales of the NIAS within IBD men and women: Picky Eating (Question 1–3), Appetite (reflecting a lack of interest in eating) (Question 4–6), and Fear (related to concerns about negative consequences) (Question 7–9). Each of these subscales can have a score ranging from 0 to 15 points, thus indicating the severity level in each area, with higher scores on the scale being indicative of a greater presence of symptoms in the respective subscale. In the further analysis the subscales are referenced as *picky*, *appetite*, and *fear*. The proposed cutoffs for clinical concern in the subscales were used. A score of ten or higher on the picky and fear subscales, or a score of nine or higher on the appetite subscale, indicated a positive AFRID risk screening [47].

### Statistical analyses

Statistical analysis was conducted using SPSS software, version 29.0.2.0 (SPSS, IBM, Armonk, NY), with additional visualization conducted in GraphPad PRISM, version 10.3.0 (GraphPad Software, Boston, Massachusetts, USA). The normality of the distribution of the data was determined using the Shapiro-Wilk test. Apart from directional hypothesis test via Pearson correlation (r) and unless otherwise stated, significance levels are two-sided. The Kaiser-Meyer-Olkin (KMO) test and Bartlett’s test of sphericity were employed to evaluate the adequacy of the data for exploratory factor analysis (EFA). The Mann Whitney U test and Fisher’s exact test were used in order to facilitate a between-group comparison. Spearman’s rank correlation coefficient (ρ) was used to analyze the relationship between NIAS and NIAS subscales with demographic and disease-specific variables, as well as questionnaire scores.

### Validity

The content validity of the translated NIAS German items was assessed based on the expert judgment method [48]. Meanwhile, construct validity was evaluated via hypothesis testing and exploratory factor analysis. The following hypothesis was put forward: the NIAS would show an inverse correlation with the FR-QoL-29. As was the case in the original NIAS validation study, the VSI was employed, and thusly a hypothesis was put forward that there would be a positive correlation between the NIAS and the VSI. Furthermore, it is suggested that the overall disease-related QoL, as measured by the SHS, will correlate with the NIAS.

### Reliability

Cronbach’s alpha was used to evaluate the internal consistency of the German version of the NIAS. Furthermore, the reliability was evaluated by means of a follow-up questionnaire, which was administered to a sample of *n* = 99 participants. The follow up was conducted on average four weeks after initial study baseline visit. In order to assess the test-retest reliability of the data the Pearson correlation coefficient was used to measure the linear relationship, and the intraclass correlation coefficient (ICC) was used as a measure of agreement, to assess the reliability of the NIAS scores. As an additional quality criterion, the potential ceiling and floor effects of the NIAS were evaluated.

### Study size

A priori sample size estimation for factor analysis specified the inclusion of ≥ 45 individuals to assess the validity and reliability of the translation.

#### Bias

Prior to study inclusion, patients were asked whether they had recently initiated or were planning to initiate a change in dietary regime. It was deemed essential to eliminate the potential introduction of bias in the analysis, and consequently, a confirmation response was a study exclusion criterion as was a diagnosed eating disorder. However, it is well documented that eating disorders frequently remain undetected for extended periods prior to diagnosis [49, 50]. Therefore, it cannot be ruled out that patients who are exhibiting classic eating disorders yet lack a diagnosis, were also included in the study. Furthermore, there is a paucity of data available on new forms of disordered eating that are yet to be indexed, such as orthorexia. However, it has been suggested that patients with IBD are particularly vulnerable to this particular form of disordered eating [51].

## Results

### Study population

A total of *n* = 235 patients with IBD were included in this analysis (women: *n* = 117 [49.8%]). Baseline demographic is well balanced between the sexes. Median age was 37 years for men and 40 years for women (*p* = 0.0240). Median disease duration was 11 years for men and 12 years for women (*p* = 0.601). The majority of men and women were in remission, and while a percentual difference in remission status (men: 76.3%; women: 67.8%) was observed, this was not significant (*p* = 0.114). In addition, women reported slightly less current ADT (Advanced Drug Therapy, such as biologic agents and small molecules) (79.5% to men: 86.4%) and corticosteroid treatment (8.5% to men: 12.7%), however this was not significant (ADT: *p* = 0.169; corticosteroids: *p* = 0.398). While a skewed distribution of disease entity was observed overall, it was well balanced between the sexes (men with CD: 62.7%; women with CD: 63.2%; *p* = 0.999). **[**Table 1: Baseline characteristics].

**Table 1:** Baseline characteristics

Demographic data		Men	Women	*p*_sex_
Entity [n(%)]	Crohn’s disease	74 (62.7%)	74 (63.2%)	0.999
	Ulcerative colitis	44 (37.3%)	43 (36.8%)	
CD Location [n(%)]	L1	18 (25%)	17 (23.6%)	0.852
	L2	10 (13.9%)	14 (19.4%)	
	L3	40 (55.6%)	36 (50%)	
	L4	4 (5.6%)	5 (6.9%)	
CD Behavior [n(%)]	B1	27 (36.5%)	36 (48.6%)	0.246
	B2	16 (21.6%)	15 (20.3%)	
	B3	29 (39.2%)	19 (25.7%)	
	B2 + B3	2 (2.7%)	4 (5.4%)	
UC Extent [n(%)]	E1	5 (14.3%	4 (12.5%)	0.530
	E2	11 (31.4%)	7 (21.9%)	
	E3	19 (54.3%)	19 (59.4%)	
Current corticosteroid [n(%)]		15 (12.7%)	10 (8.5%)	0.398
Current ADT [n(%)]		102 (86.4%)	93 (79.5%)	0.169
Currently in remission [n(%)]		90 (76.3%)	78 (67.8%)	0.114
Prior IBD surgery [n(%)]		31 (26.3%)	34 (29.1%)	0.664
Current use of antidepressant [n(%)]		6 (5.1%)	12 (10.3%)	0.150
Current or former smoker [n(%)]		51 (43.2%)	55 (47%)	0.601
Disease duration [Md (IQR)] (yrs)		11 [6–18]	12 [6–20]	0.601
Age [Md (IQR)] (yrs)		37 [26–54]	40 [29–55]	0.240
Vitamin D [Md (IQR)] (µg/l)		29 [21–36]	31 [24–40]	0.079
Fecal Calprotectin [Md (IQR)] (µg/dl )		108 [37.8–467]	86.7 [30–274.5]	0.362
CRP [Md (IQR)] (mg/l)		0.2 [0–1.4]	0.5 [0–2.4]	0.119
BMI [Md (IQR)] (kg/m^2^)		23.7 [21.5–26.6]	23.8 [20.8–26.6]	0.703

Variables are expressed as total and percentage [n(%)] or median and interquartile range [MD (IQR)]. Significance values were determined via fisher’s exact test or Mann Whitney U test. Significance levels are two-sided

CD – Crohn‘s disease; UC – ulcerative colitis; ADT – advanced drug therapy; IBD – inflammatory bowel disease; CRP – C-reactive protein; BMI – body mass index

### Translation and cross-cultural adaptation

The translation and subsequent cross-cultural adaptation of the questionnaire into German comprised a multi-stage process, including a blinded backwards translation. Despite minor adjustments to sentence structure, the final German version of the NIAS is substantively equivalent to its original counterpart. In accordance with the process of similar projects [41] the translation of the Likert scale labels within the German context was subjected to meticulous review. In lieu of adopting a direct, literal translation of “Strongly agree” and “Strongly disagree”, the German responses of “Stimme voll und ganz zu” (English translation: Completely agree) and “Stimme überhaupt nicht zu” (English translation: Completely disagree) were selected. This decision is consistent with established practice, as these formulations are widely recognized and frequently implemented in German standardized questionnaires to ensure clarity.

### Validity

The requirements for factor analysis were met with the inclusion of a minimum of 45 enrolled study participants. This was further substantiated by the result of the KMO test (0.788) while in addition Bartlett’s test of sphericity was significant (χ2 = 489; *p* < 0.001), thus indicating that the data was suitable for EFA. Three factors with eigenvalue > 1 were identified via factor analysis, which accounted for a total variance of 74.94%. Overall, a total of 41.54%% of the observed variance was attributed to factor 1. [Table 2: Exploratory factor analysis for items of the German version of the Nine Item Avoidant/Restrictive Food Intake disorder screen (NIAS)]

**Table 2:** Exploratory factor analysis for items of the German version of the Nine Item Avoidant/Restrictive Food Intake disorder screen (NIAS)

NIAS German item	Question	Factor 1
1	I am a picky eater	0.431
2	I dislike most of the foods that other people eat	0.465
3	The list of foods that I like and will eat is shorter than the list of foods I won’t eat	0.523
4	I am not very interested in eating; I seem to have a smaller appetite than other people	0.683
5	I have to push myself to eat regular meals throughout the day, or to eat a large enough amount of food at meals	0.747
6	Even when I am eating a food I really like, it is hard for me to eat a large enough volume at meals	0.785
7	I avoid or put off eating because I am afraid of GI discomfort, choking, or vomiting	0.759
8	I restrict myself to certain foods because I am afraid that other foods will cause GI discomfort, choking, or vomiting	0.616
9	I eat small portions because I am afraid of GI discomfort, choking, or vomiting	0.683
	Eigenvalue	3.74
	% of Variance	41.54

Exploratory factor analysis for items of the German version of the Nine Item Avoidant/Restrictive Food Intake disorder screen

NIAS - Nine Item Avoidant/Restrictive Food Intake disorder screen; GI – gastrointestinal

The absence of ceiling and floor effects was evident in the results: of 195 completed screeners the minimum score of 0 was reached four times (2.1%). Meanwhile, no participants attained the maximum score of 45.

Validity was further assessed by hypothesis testing for the hypotheses formulated in advance. The results showed a positive correlation between NIAS score and the VSI score (*p* < 0.001); and an inverse correlation for NIAS score and the FR-QoL-29 German score (*p* < 0.001). In addition, a positive correlation was observed for (a) SHS score (*p* < 0.001), (b) HADS anxiety score (*p* < 0.001), (c) HADS depression score (*p* < 0.001), (d) sex (*p* = 0.012), and (e) antidepressant usage (*p* = 0.016). An inverse correlation was observed for (a) FACIT fatigue score (*p* < 0.001), (b) disease duration (*p* = 0.040), (c) height (*p* < 0.001) and (d) weight (*p* = 0.003). However, no correlation was observed for BMI (*p* = 0.144) and age (*p* = 0.799). [Table 3: One-sided results of directional hypothesis testing.]

**Table 3:** One-sided results of directional hypothesis testing

Correlation factors	*n*	Pearson correlation (*r*)	*p*
Sex	128	0.199	**0.012**
Entity	128	-0.14	0.439
Remissionstatus	128	0.173	**0.025**
Antidepressants	128	0.189	**0.016**
IBD surgery	128	0.007	0.470
Age	128	0.023	0.399
Height	128	-0.276	**< 0.001**
Weight	128	-0.240	**0.003**
BMI	128	-0.130	0.072
Disease duration	128	-0.155	**0.040**
Vitamin D	123	0.093	0.154
CRP	127	0.059	0.254
Fecal Calprotectin	113	0.113	0.116
SHS	124	0.426	**< 0.001**
FACIT Fatigue	128	-0.456	**< 0.001**
HADS Anxiety	128	0.321	**< 0.001**
HADS Depression	128	0.332	**< 0.001**
VSI	128	0.495	**< 0.001**
sQ-HPF	128	-0.028	0.375
FR-QoL-29	128	-0.542	**< 0.001**
MEDAS	128	0.101	0.129

Correlation of demographic, health and disease specific factors with the NIAS score - One-sided results of directional hypothesis testing

NIAS - Nine Item Avoidant/Restrictive Food Intake disorder screen; IBD – inflammatory bowel disease; BMI – body mass index; CRP – c-reactive protein; SHS – short health scale; FACIT Fatigue - Functional Assessment of Chronic Illness Therapy, Fatigue Scale; HADS – Hospital Anxiety and Depression Scale; VSI - Visceral Sensitivity Index; sQ-HPF - short screening questionnaire of highly processed food consumption; FR-QoL-29 – Food-related Quality of Life; MEDAS – Mediterranean diet adherence screener

### Reliability

The NIAS German demonstrated high internal consistency, as indicated by a Cronbach’s α coefficient of 0.810. Furthermore, the deletion of individual items resulted in a decline in the Cronbach’s α coefficient, ranging from 0.779 to 0.808. Thus, no single item was deleted. Pearson correlation coefficient analysis demonstrated excellent test-retest reliability between the initial baseline NIAS score and the subsequent follow-up NIAS score (*p* < 0.001). A significant single item correlation was additionally identified between the initial and follow-up time points for all German NIAS items (*p* < 0.001). Lastly, the calculated intraclass correlation coefficient of 0.896 [95% CI: 0.846–0.931] further substantiated the reliability of the German questionnaire version. [Table 4: Results for internal consistency analysis and test-retest reliability].

**Table 4:** Results for internal consistency analysis and test-retest reliability

NIAS German item	Corrected item-total score correlation	Cronbach’s Alpha if item deleted	Test-retest reliability
1	0.388	0.808	0.756*
2	0.439	0.799	0.722*
3	0.472	0.797	0.749*
4	0.534	0.789	0.619*
5	0.569	0.783	0.798*
6	0.620	0.779	0.634*
7	0.606	0.779	0.601*
8	0.461	0.797	0.627*
9	0.531	0.788	0.720*
		**No of items**	9
		**Cronbach’s Alpha**	0.810
**Intraclass correlation coefficient [95% CI]**			0.896 [0.846–0.931]

Results for internal consistency analysis and test-retest reliability

NIAS - Nine Item Avoidant/Restrictive Food Intake disorder screen

### Sex-differences in the NIAS and its subscales

**Fig. 1:**
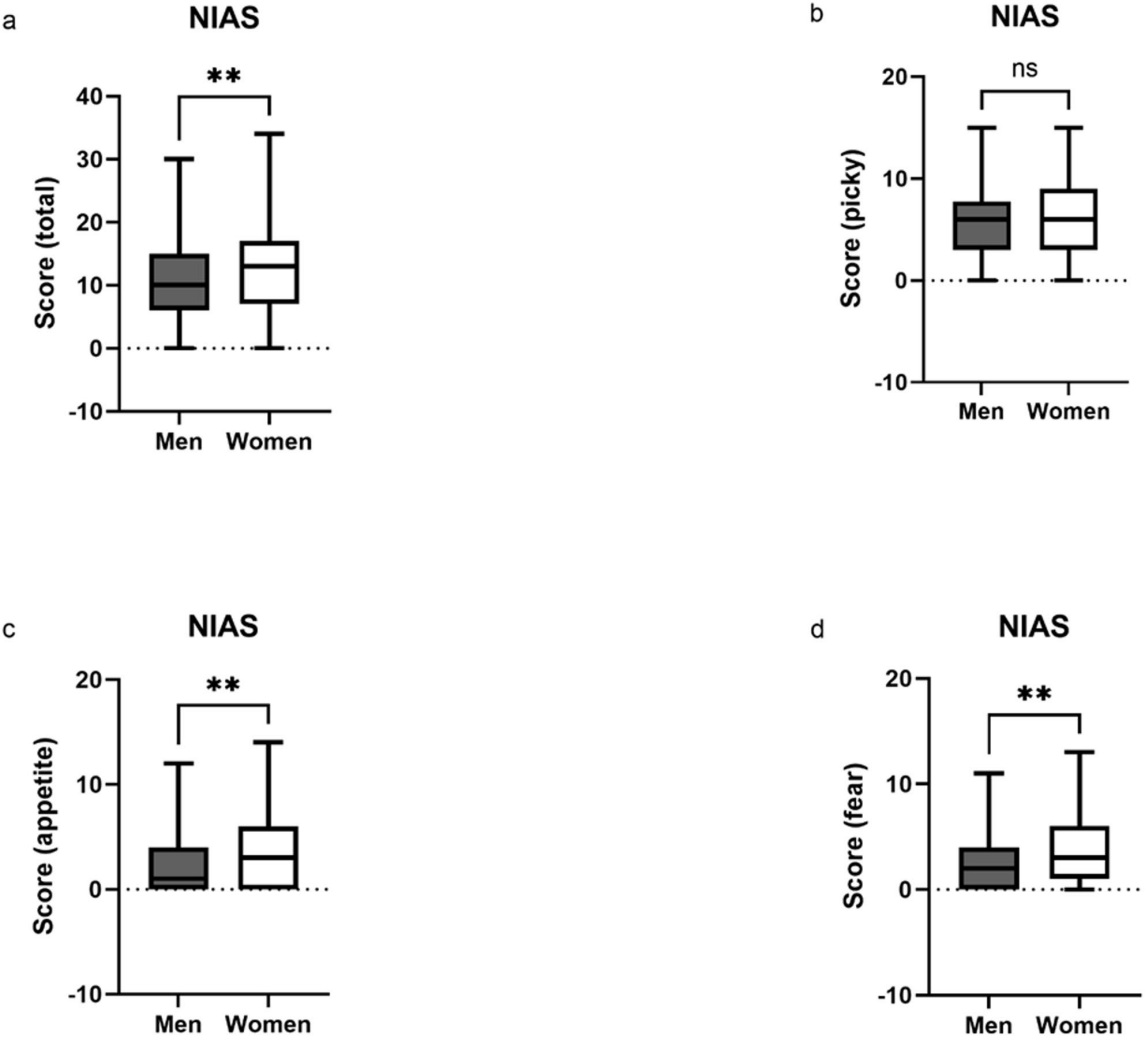
**a**-**d** Nonparametric (Mann-Whitney U) comparison of **a** overall NIAS score between men and women with IBD (*p* = 0.007); **b** NIAS – picky subscale between men and women with IBD (*p* = 0.461); **c** NIAS – appetite subscale between men and women with IBD (*p* = 0.009); **d** NIAS – fear subscale between men and women with IBD (*p* = 0.004). Significance levels are indicated in figures as one asterisk for *p* = 0.05, two for *p* = 0.01, and three for *p* < 0.001. NIAS - NIAS - Nine Item Avoidant/Restrictive Food Intake disorder screen; IBD – inflammatory bowel disease; ns – not significant

To investigate possible differences between the sexes in the scoring for the NIAS and its subscales Mann Whitney U test was employed. The results showed that women with IBD overall scored significantly higher, indicating a greater likelihood and severity of ARFID (Md [IQR]: women = 13 [7–17]; men = 10 [6–15]; *p* = 0.007). This was also true for the subscales *Appetite* (Md [IQR]: women: 3 [0–6]; men = 1 [0–4]; *p* = 0.009), and *Fear* (Md [IQR]: women = 3 [1–6]; men = 2 [0–4]; *p* = 0.004), but not for *Picky* (Md [IQR]: women = 6 [3–9]; men = 6 [3–8]; *p* = 0.461). **[**Figure 1a-d**]**

### Association between NIAS and food-related QoL in patients with IBD

In addition, possible sex-specific patterns in correlation of IBD specific food related QoL and ARFID symptoms, as screened by the NIAS were explored. Two-sided spearman correlation showed that the FR-QoL-29 and the overall NIAS score had an inverse correlation for men (*p* < 0.001) and women (*p* < 0.001). This was also observed for the NIAS subscales *Appetite* (men: *p* < 0.001; women: *p* = 0.018) and *Fear* (men: *p* < 0.001; women: *p* < 0.001). The correlation of FR-QoL-29 and the NIAS subscale *Picky* however was only significant for IBD men (*p* = 0.039) but not women (; *p* = 0.104). **[**Table 5: Results of two-sided sex-specific spearman correlation of FR-QoL-29 with NIAS.].

**Table 5:** Results of two-sided sex-specific spearman correlation of FR-QoL-29 with NIAS

	Food-related QoL			
	Sex	*n*	Spearman’s Rho (ρ)	*p*
NIAS	Men	59	-0.575	**< 0.001**
	Women	69	-0.478	**< 0.001**
NIAS - Picky	Men	59	-0.269	**0.039**
	Women	69	-0.198	0.104
NIAS - Appetite	Men	59	-0.468	**< 0.001**
	Women	69	-0.284	**0.018**
NIAS - Fear	Men	59	-0.566	**< 0.001**
	Women	69	-0.561	**< 0.001**

Results of two-sided sex-specific spearman correlation of FR-QoL-29 with NIAS

NIAS - NIAS - Nine Item Avoidant/Restrictive Food Intake disorder screen; FR-QoL-29 – Food-related QoL

### Sex- and entity-specific correlation patterns in avoidant and restrictive eating behavior

To obtain a more nuanced investigation of ARFID symptoms, as screened by the NIAS in this heterogeneous population, additional correlation patterns were examined using a stratified dataset, with the analysis divided by sex and entity. As demonstrated in Table 6, the results of a two-sided, sex- and entity-specific Spearman correlation showed distinctive differences.

In relation to the previously utilized scores for hypothesis testing, a significant correlation was observed between the visceral sensitivity index (VSI) and the overall NIAS score, as well as the Fear subscale, in women with CD. (NIAS overall: *p* < 0.001; Fear: *p* < 0.001). However, this correlation was not observed in women with UC (NIAS overall: *p* = 0.179. Fear: *p* = 0.271). Conversely, men with IBD exhibited an even more pronounced correlation with visceral sensitivity (NIAS overall: CD: *p* = 0.005; UC: *p* = 0.037. Appetite: CD: *p* = 0.010; UC: *p* = 0.059. Fear: CD: *p* = 0.009; UC: *p* = 0.003). Furthermore, the correlation between IBD food-related QoL and the overall NIAS score, as well as the Fear subscale, was observed to be significant across both entities for both men and women with IBD (NIAS overall: men with CD: *p* < 0.001; women with CD: *p* = 0.001; men with UC: *p* < 0.001; women with UC:; *p* = 0.009. Fear: men with CD: *p* < 0.001; women with CD: *p* < 0.001; men with UC: *p* = 0.003; women with UC: *p* = 0.011.). While no correlation was observed on the Picky subscale, men and women with CD and men with UC exhibited significant correlations between IBD food-related QoL and the Appetite subscale (Appetite: men with CD: *p* = 0.005; women with CD: *p* = 0.031; men with UC: *p* = 0.008; women with UC: *p* = 0.109.).

A substantial correlation was observed in relation to the objective disease parameter, fecal calprotectin. However, this correlation was exclusively evident in men with UC on the Appetite subscale (*p* = 0.029).) This correlation was not detected in women with UC or in men and women in the CD cohort.

Concurrently, a substantial correlation between the consumption of highly processed food (HPF), as determined by the short screening questionnaire of highly processed food consumption (sQ-HPF), was exclusively discernible among men with CD and UC (NIAS overall: CD: *p* = 0.075; UC: *p* = 0.023. Picky: CD: *p* = 0.020; UC: *p* = 0.009). No such correlation was evident among their female counterparts (NIAS overall: CD: *p* = 0.450; UC: *p* = 0.802. Picky: CD: *p* = 0.520; UC: *p* = 0.975). Overall, no significant correlations within the sexes and entities were observed for serum-25(OH) D3, BMI, and age. [Table 6: Results of two-sided sex- and entity-specific spearman correlation].

**Table 6:** Results of two-sided sex- and entity-specific spearman correlation

	Sex	NIAS								NIAS - Picky						NIAS - Appetite						NIAS - Fear					
		CD				UC				CD			UC			CD			UC			CD			UC		
		n	Spearman’s Rho (ρ)	p		n	Spearman’s Rho (ρ)	p		Spearman’s Rho (ρ)	p		Spearman’s Rho (ρ)	p		Spearman’s Rho (ρ)	p		Spearman’s Rho (ρ)	p		Spearman’s Rho (ρ)	p		Spearman’s Rho (ρ)	p	
SHS	Men	36	0.355	**0.034**		20	0.570	**0.009**		0.319	0.058		0.253	0.282		0.204	0.234		0.553	**0.011**		0.224	0.189		0.525	**0.017**	
	Women	50	0.313	**0.027**		18	0.546	**0.019**		0.101	0.485		0.379	0.121		0.272	0.056		0.481	**0.043**		0.408	**0.003**		0.515	**0.029**	
Facit-Fatigue	Men	38	-0.478	**0.002**		21	-0.488	**0.025**		-0.448	**0.005**		-0.298	0.189		-0.425	**0.008**		-0.483	**0.027**		-0.140	0.403		-0.434	**0.049**	
	Women	51	-0.261	0.065		18	-0.500	**0.035**		-0.130	0.364		-0.298	0.229		-0.163	0.253		-0.626	**0.005**		-0.328	**0.019**		-0.417	0.085	
VSI	Men	38	0.449	**0.005**		21	0.458	**0.037**		0.289	0.078		0.052	0.822		0.411	**0.010**		0.419	0.059		0.417	**0.009**		0.615	**0.003**	
	Women	51	0.470	**< 0.001**		18	0.331	0.179		0.212	0.135		0.196	0.435		0.226	0.110		0.362	0.140		0.585	**< 0.001**		0.274	0.271	
sQ-HPF	Men	38	0.293	0.075		21	-0.494	**0.023**		0.377	**0.020**		-0.558	**0.009**		0.233	0.179		-0.081	0.727		0.046	0.783		-0.187	0.416	
	Women	51	-0.108	0.450		18	0.063	0.802		-0.092	0.520		0.008	0.975		0.023	0.873		0.155	0.539		-0.105	0.464		0.020	0.938	
FR-QoL-29	Men	38	-0.542	**< 0.001**		21	-0.687	**< 0.001**		-0.299	0.069		-0.318	0.161		-0.446	**0.005**		-0.559	**0.008**		-0.513	**< 0.001**		-0.609	**0.003**	
	Women	51	-0.436	**0.001**		18	-0.594	**0.009**		-0.158	0.268		-0.431	0.074		-0.302	**0.031**		-0.391	0.109		-0.546	**< 0.001**		-0.584	**0.011**	
Fecal Calprotectin	Men	36	0.025	0.883		18	0.348	0.157		0.034	0.842		-0.102	0.687		0.028	0.871		0.516	**0.029**		0.010	0.952		0.388	0.112	
	Women	43	0.194	0.212		16	-0.213	0.429		0.242	0.118		-0.430	0.097		0.039	0.805		-0.057	0.834		0.117	0.457		-0.116	0.668	
Vitamin D	Men	35	-0.119	0.497		21	0.307	0.177		-0.131	0.452		0.257	0.261		0.060	0.733		0.124	0.591		-0.003	0.987		0.098	0.673	
	Women	49	0.274	0.056		18	-0.204	0.418		0.251	0.082		-0.102	0.687		0.178	0.220		-0.362	0.140		0.161	0.269		-0.081	0.748	
BMI	Men	38	-0.168	0.313		21	-0.110	0.636		-0.040	0.813		-0.156	0.498		-0.128	0.443		0.047	0.840		-0.283	0.085		0.037	0.874	
	Women	51	-0.072	0.614		18	-0.366	0.135		0.055	0.702		-0.257	0.304		-0.111	0.438		-0.384	0.116		-0.039	0.785		-0.373	0.128	
Age	Men	38	-0.102	0.542		21	-0.044	0.849		-0.062	0.711		0.037	0.873		-0.138	0.409		-0.073	0.754		-0.105	0.530		0.084	0.716	
	Women	51	0.126	0.379		18	-0.339	0.169		0.228	0.107		-0.030	0.907		0.007	0.960		-0.352	0.153		0.135	0.346		-0.380	0.120	
Disease duration	Men	38	-0.185	0.267		21	-0.243	0.289		-0.105	0.529		-0.323	0.154		-0.111	0.508		-0.200	0.385		-0.323	**0.048**		0.095	0.683	
	Women	51	0.004	0.976		18	-0.701	**0.001**		0.106	0.460		-0.302	0.223		-0.068	0.634		-0.634	**0.005**		-0.010	0.945		-0.772	**< 0.001**	

Results of two-sided sex- and entity-specific spearman correlation

NIAS - Nine Item Avoidant/Restrictive Food Intake disorder screen; SHS – short health scale; FACIT Fatigue - Functional Assessment of Chronic Illness Therapy, Fatigue Scale; VSI - Visceral Sensitivity Index; sQ-HPF - short screening questionnaire of highly processed food consumption; FR-QoL-29 – Food-related Quality of Life; CD – Crohn’s disease; UC – ulcerative colitis

## Discussion

The present analysis sought to validate the novel German translation of the Nine Items Avoidant/Restrictive Food Intake Disorder Screen (NIAS), a tool developed for an initial screening of ARFID [17], in a heterogeneous, multicentric cohort of patients with IBD. In this context, sex- and gender-specific differences in ARFID symptoms, as screened by the NIAS, were also explored.

The German version of the NIAS demonstrated high internal consistency, as indicated by a Cronbach’s α coefficient of 0.81. This is in line with other cross-cultural adaptations and translation studies of the NIAS which reported for example Cronbach’s α coefficient of 0.84 [19] or 0.75 [20]. Consequently, we introduce the NIAS German as a valid and reliable tool to screen for ARFID in men and women with IBD.

The analysis into sex-specific differences in ARFID tendencies as screened by the NIAS and its distinct correlations with IBD disease associated factors indicates distinct correlation patterns for men and women with IBD, with women being more likely to score higher on the NIAS, particularly those with a shorter disease duration. Consequently, these findings may suggest a potential for more disease-related adaptive eating behaviors in men with IBD, which aligns with previous research findings [23]. Overall, while aspects that also overlap with ARFID, including anxiety and eating disorders, are more commonly observed in women with IBD, notable manifestations are observed in men in contrast, particularly in relation to sensory-related food refusal as identified by the Picky subscale. It can be posited that the significant correlation observed between the sQ-HPF and both the overall NIAS score in men with UC and the picky score in men with UC and men with CD may be indicative of sensory-related food refusal as well as a lack of food literacy. Surprisingly, the correlation was found to be positive in men with CD and inverse in men with UC. This finding may be indicative of disparate underlying motivations. Food refusal, characterized by a reluctance to consume particular foods, is frequently observed in neurodiverse populations. This refusal can be attributed to sensory sensitivities, which manifest as heightened reactions to various sensory stimuli, including texture, taste, smell, temperature, color, and appearance [52–54]. Therefore, it could be posited that a propensity for HPFs may be attributable to the dependable textures and flavors inherent in highly processed food.

Overall, the fact that a positive correlation was identified between the NIAS score and the Visceral Sensitivity Index (VSI) score suggests that patients with IBD who experience heightened visceral anxiety are more prone to manifesting ARFID-like symptoms, as screened by the NIAS. Conversely, a negative correlation was observed between the NIAS score and food-related QoL. These correlations of disease-related scores with the NIAS suggest that the measured scores may not necessarily be indicative of personality traits such as anxiety, but rather specific for disease-related anxiety. The experience of chronic illnesses can result in a sense of loss of control for those affected, particularly during the initial diagnosis phase and as the illness progresses [55, 56]. Modifiable lifestyle and health factors, including smoking, alcohol consumption, lack of exercise and poor nutrition, can be influenced by affected individuals, thereby enabling them to exert a degree of control over their health [57] and possibly gaining a better QoL [58]. However, this might also have negative implications such as leading to restrictive or avoidant food habits [59–61]. In the context of IBD especially, the tendency of patients to engage in restrictive eating behaviors as a means of modifying the severity and symptoms of the disease as well as preventing flares, has been well-documented [11, 12]; with a special focus in recent years on the interconnection of ARFID and IBD [21, 62]. Further in-depth analyses are necessary to determine the direction of the influence. Moreover, a recent study found that ARFID risk in patients with IBD is associated with poor food literacy [63].

The overall results of this study underscore the necessity for a more personalized approach to the treatment of IBD, encompassing heightened awareness of ARFID symptoms and the integration of the NIAS as a fast, screening instrument. Sex-and gender-specific differences within the NIAS and its distinct correlations with further clinical aspects in IBD highlight the importance of differential analyses within this field to elucidate heterogeneity and enable individualized patient-centered screening pipelines and recommendations. Furthermore, the results of this study suggest that dietary consultations especially for patients newly diagnosed with IBD, but also in general during the course of the disease, may be beneficial in preventing the manifestation of eating disorders. The present study has several implications for the management of patients with IBD, including the need for increased awareness of ARFID symptoms among healthcare providers, the use of the NIAS score as a screening tool, and the consideration of sex- and entity-specific differences in the manifestation of ARFID symptoms. It is recommended that subsequent studies endeavor to replicate the findings of this study, and furthermore to explore the relationship between ARFID symptoms and IBD activity in larger cohorts of patients. Furthermore, such studies should investigate the effectiveness of targeted interventions in the prevention of the manifestations of eating disorders in patients with IBD. In addition, novel phenomena such as orthorexia, which show a high prevalence in patients with IBD [51], should also be considered in future research. Finally, it is important to note that patients should receive dietary support at the point of diagnosis and throughout the entire course of the disease.

Despite evident merits of this study, such as the multicentric approach and comprehensive analysis for validity and reliability, some limitations must be acknowledged. For one, the present analysis does not permit statements regarding possible influencing factors and directions. Moreover, study participants were not screened for malnutrition. As ARFID patients frequently present with malnutrition, the incorporation of this element would have constituted a valuable addition by facilitating an objective verification of the NIAS score and its effects on nutritional status. A key limitation of this study is the absence of a gold-standard diagnostic assessment for ARFID (e.g., structured clinical interview). As a result, we were unable to evaluate criterion validity or determine the sensitivity and specificity of the NIAS German in detecting ARFID cases. Consequently, conclusions regarding its effectiveness as a screening tool should be interpreted with caution. Future studies should incorporate clinical diagnostic procedures to assess the instrument’s diagnostic accuracy and establish appropriate cut-off scores in German-speaking populations with IBD.

In conclusion, the German version of the NIAS demonstrated good reliability and initial evidence supporting its use as a screening instrument in German-speaking men and women with IBD. The results of the present analysis suggest that women with IBD are at elevated risk of developing restrictive or avoidant eating habits while men with IBD may show tendencies for a more adaptive eating behavior. Further underscoring the necessity of sex-specific assessments in patients with IBD.

## Data Availability

All data supporting the findings of this study are available within the paper.

## Abbreviations

ARFID: Avoidant/restrictive food intake disorder
BMI: Body mass index
CD: Crohn’s disease
ED: Eating disorder
EFA: Exploratory factor analysis
FACIT: Fatigue–Functional Assessment of Chronic Illness Therapy–Fatigue Scale
FR: QoL–food–related quality of life
GI: Gastrointestinal
HADS: Hospital anxiety and depression scale
HPF: Highly Processed Food
IBD: Inflammatory bowel disease
KMO: Kaiser–Meyer–Olkin
MEDAS: Mediterranean diet adherence screener
NIAS: Nine Item Avoidant/Restrictive Food Intake Disorder Screen
QoL: Quality of life
SHS: Short health scale
sQ: HPF–screening questionnaire of highly processed food consumption
UC: Ulcerative colitis
VSI: Visceral sensitivity index

## References

[1] Colombel J-F, Shin A, Gibson PR. AGA Clinical Practice Update on Functional Gastrointestinal Symptoms in Patients With Inflammatory Bowel Disease: Expert Review. Clin Gastroenterol Hepatol. 2019;17(3):380–e901. 30099108 10.1016/j.cgh.2018.08.001 PMC6581193

[2] Adolph TE, Zhang J. Diet fuelling inflammatory bowel diseases: preclinical and clinical concepts. Gut. 2022;71(12):2574–86. 36113981 10.1136/gutjnl-2021-326575 PMC9664119

[3] Levine A, Sigall Boneh R, Wine E. Evolving role of diet in the pathogenesis and treatment of inflammatory bowel diseases. Gut. 2018;67(9):1726–38. 29777041 10.1136/gutjnl-2017-315866

[4] Cohen AB, Lee D, Long MD, Kappelman MD, Martin CF, Sandler RS, et al. Dietary patterns and self-reported associations of diet with symptoms of inflammatory bowel disease. Dig Dis Sci. 2013;58(5):1322–8. 22923336 10.1007/s10620-012-2373-3 PMC3552110

[5] Godala M, Gaszyńska E, Durko Ł, Małecka-Wojciesko E. Dietary Behaviors and Beliefs in Patients with Inflammatory Bowel Disease. J Clin Med. 2023;12(10):3455. 37240560 10.3390/jcm12103455 PMC10219397

[6] Manski S, Noverati N, Policarpo T, Rubin E, Shivashankar R. Diet and Nutrition in Inflammatory Bowel Disease: A Review of the Literature. Crohns Colitis 360. 2024;6(1):otad077. 38213632 10.1093/crocol/otad077 PMC10782214

[7] Guthrie E, Jackson J, Shaffer J, Thompson D, Tomenson B, Creed F. Psychological disorder and severity of inflammatory bowel disease predict health-related quality of life in ulcerative colitis and Crohn’s disease. Am J Gastroenterol. 2002;97(8):1994–9. 12190166 10.1111/j.1572-0241.2002.05842.x

[8] Sarwan N, Jurawan R, Singh R, Chattu VK. An Exploratory Study of Inflammatory Bowel Disease and the Psychosocial Factors Affecting Health-Related Quality of Life. Med Sci (Basel). 2019;7(2). 10.3390/medsci7020018 PMC6409541 30691020

[9] Casanova MJ, Chaparro M, Molina B, Merino O, Batanero R, Dueñas-Sadornil C, et al. Prevalence of Malnutrition and Nutritional Characteristics of Patients With Inflammatory Bowel Disease. J Crohns Colitis. 2017;11(12):1430–9. 28981652 10.1093/ecco-jcc/jjx102

[10] Pueschel L, Wedemeyer H, Lenzen H, Wiestler M. Sex Differences Outweigh Dietary Factors in Food-Related Quality of Life in Patients with Inflammatory Bowel Disease. Nutrients. 2025;17(7):1114. 40218872 10.3390/nu17071114 PMC11990271

[11] Day AS, Yao CK, Costello SP, Andrews JM, Bryant RV. Food-related quality of life in adults with inflammatory bowel disease is associated with restrictive eating behaviour, disease activity and surgery: A prospective multicentre observational study. J Hum Nutr Diet. 2022;35(1):234–44. 34008222 10.1111/jhn.12920

[12] Day AS, Yao CK, Costello SP, Andrews JM, Bryant RV. Food avoidance, restrictive eating behaviour and association with quality of life in adults with inflammatory bowel disease: A systematic scoping review. Appetite. 2021;167:105650. 34391842 10.1016/j.appet.2021.105650

[13] Coglan L, Otasowie J. Avoidant/restrictive food intake disorder: What do we know so far? BJPsych Adv. 2019;25:90–8.

[14] Culbert KM, Sisk CL, Klump KL. A Narrative Review of Sex Differences in Eating Disorders: Is There a Biological Basis? Clin Ther. 2021;43(1):95–111. 33375999 10.1016/j.clinthera.2020.12.003 PMC7902379

[15] Archibald T, Bryant-Waugh R. Current evidence for avoidant restrictive food intake disorder: Implications for clinical practice and future directions. JCPP Adv. 2023;3(2):e12160. 37753149 10.1002/jcv2.12160 PMC10519741

[16] Kambanis PE, Thomas JJ. Advancing the Science of Avoidant/Restrictive Food Intake Disorder (ARFID): Six Key Questions. Int J Eat Disord. 2025;58(6):1001–7. 40108823 10.1002/eat.24418 PMC12264816

[17] Zickgraf HF, Ellis JM. Initial validation of the Nine Item Avoidant/Restrictive Food Intake disorder screen (NIAS): A measure of three restrictive eating patterns. Appetite. 2018;123:32–42. 29208483 10.1016/j.appet.2017.11.111

[18] Van Ouytsel P, Mikhael-Moussa H, Mion F, Roman S, Gourcerol G, Marion-Letellier R, et al. Translation of the nine item avoidant/restrictive food intake disorder screen (NIAS) questionnaire in French (NIAS-Fr). Neurogastroenterol Motil. 2024;36(4):e14757. 38308088 10.1111/nmo.14757

[19] Medina-Tepal KA, Vazquez-Arevalo R, Trujillo-ChiVacuán EM, Zickgraf HF, Mancilla-Díaz JM. Cross-cultural adaptation and validation of the Nine Item ARFID Screen (NIAS) in Mexican youths. Int J Eat Disord. 2023;56(4):721–6. 36268632 10.1002/eat.23832

[20] Kaşak M, Öğütlü H, Doğan U, Zickgraf HF, Türkçapar MH. Psychometric properties of the nine-item avoidant/restrictive food intake disorder screen (NIAS) in Turkish adolescents. J Eat Disord. 2024;12(1):105. 39060938 10.1186/s40337-024-01066-0 PMC11282626

[21] Simons M, Issokson K. From Food Fears to Food Freedom: How Do We Best Manage Restrictive Eating in Inflammatory Bowel Disease? Crohns Colitis 360. 2025;7(2):otaf019. 40290581 10.1093/crocol/otaf019 PMC12022839

[22] Tshikudi DM, Bernstein CN, Mishra S, Ghia JE, Armstrong HK. Influence of biological sex in inflammatory bowel diseases. Nat Rev Gastroenterol Hepatol. 2025;22(6):415–37. 39962330 10.1038/s41575-025-01038-y

[23] Pueschel L, Kockelmann F, Kueck M, Tegtbur U, Attaran-Bandarabadi M, Bachmann O et al. Patients with Inflammatory Bowel Disease Show Fewer Sex-Related Differences in Their Dietary Behavior Than the General Population: A Qualitative Analysis. Nutrients. 2024;16(17). 10.3390/nu16172954 PMC11397495 39275270

[24] Hoek HW. Incidence, prevalence and mortality of anorexia nervosa and other eating disorders. Curr Opin Psychiatry. 2006;19(4):389–94. 16721169 10.1097/01.yco.0000228759.95237.78

[25] Striegel-Moore RH, Rosselli F, Perrin N, DeBar L, Wilson GT, May A, et al. Gender difference in the prevalence of eating disorder symptoms. Int J Eat Disord. 2009;42(5):471–4. 19107833 10.1002/eat.20625 PMC2696560

[26] Labus JS, Bolus R, Chang L, Wiklund I, Naesdal J, Mayer EA, et al. The Visceral Sensitivity Index: development and validation of a gastrointestinal symptom-specific anxiety scale. Aliment Pharmacol Ther. 2004;20(1):89–97. 15225175 10.1111/j.1365-2036.2004.02007.x

[27] Labus JS, Mayer EA, Chang L, Bolus R, Naliboff BD. The central role of gastrointestinal-specific anxiety in irritable bowel syndrome: further validation of the visceral sensitivity index. Psychosom Med. 2007;69(1):89–98. 17244851 10.1097/PSY.0b013e31802e2f24

[28] Trieschmann K, Chang L, Park S, Naliboff B, Joshi S, Labus JS, et al. The visceral sensitivity index: A novel tool for measuring GI-symptom-specific anxiety in inflammatory bowel disease. Neurogastroenterol Motil. 2022;34(9):e14384. 35478469 10.1111/nmo.14384 PMC9427691

[29] Montan I, Löwe B, Cella D, Mehnert A, Hinz A. General Population Norms for the Functional Assessment of Chronic Illness Therapy (FACIT)-Fatigue Scale. Value Health. 2018;21(11):1313–21. 30442279 10.1016/j.jval.2018.03.013

[30] Cella D, Lai JS, Chang CH, Peterman A, Slavin M. Fatigue in cancer patients compared with fatigue in the general United States population. Cancer. 2002;94(2):528–38. 11900238 10.1002/cncr.10245

[31] Yellen SB, Cella DF, Webster K, Blendowski C, Kaplan E. Measuring fatigue and other anemia-related symptoms with the Functional Assessment of Cancer Therapy (FACT) measurement system. J Pain Symptom Manage. 1997;13(2):63–74. 9095563 10.1016/s0885-3924(96)00274-6

[32] Zigmond AS, Snaith RP. The Hospital Anxiety and Depression Scale. Acta psychiatrica Scandinavica. 1983;67(6):361–70. 6880820 10.1111/j.1600-0447.1983.tb09716.x

[33] Demmer S, Kleindienst N, Hjortswang H, Thomann P, Ebert M, Reindl W, et al. Validation of the German version of the Short Health Scale - a brief, valid and reliable instrument to assess health-related quality of life in German-speaking patients with inflammatory bowel diseases. Z Gastroenterol. 2023;61(9):1207–13. 37309101 10.1055/a-1976-9971 PMC10629489

[34] Stjernman H, Grännö C, Järnerot G, Ockander L, Tysk C, Blomberg B, et al. Short health scale: a valid, reliable, and responsive instrument for subjective health assessment in Crohn’s disease. Inflamm Bowel Dis. 2008;14(1):47–52. 17828783 10.1002/ibd.20255

[35] Hjortswang H, Järnerot G, Curman B, Sandberg-Gertzén H, Tysk C, Blomberg B, et al. The Short Health Scale: a valid measure of subjective health in ulcerative colitis. Scand J Gastroenterol. 2006;41(10):1196–203. 16990205 10.1080/00365520600610618

[36] Martinez-Perez C, Daimiel L, Climent-Mainar C, Martínez-González M, Salas-Salvadó J, Corella D, et al. Integrative development of a short screening questionnaire of highly processed food consumption (sQ-HPF). Int J Behav Nutr Phys Act. 2022;19(1):6. 35073909 10.1186/s12966-021-01240-6 PMC8785596

[37] Pueschel L, Nothacker S, Wedemeyer H, Lenzen H, Wiestler M. Validation of the German Version of the Screening Questionnaire for Highly Processed Food Consumption (sQ-HPF). Aktuel Ernahrungsmed. 2025;50(05):343–53. 10.3390/jcm14113819 PMC12155798 40507581

[38] Martínez-González MA, Fernández-Jarne E, Serrano-Martínez M, Wright M, Gomez-Gracia E. Development of a short dietary intake questionnaire for the quantitative estimation of adherence to a cardioprotective Mediterranean diet. Eur J Clin Nutr. 2004;58(11):1550–2. 15162136 10.1038/sj.ejcn.1602004

[39] Hebestreit K, Yahiaoui-Doktor M, Engel C, Vetter W, Siniatchkin M, Erickson N, et al. Validation of the German version of the Mediterranean Diet Adherence Screener (MEDAS) questionnaire. BMC Cancer. 2017;17(1):341. 28521737 10.1186/s12885-017-3337-y PMC5437541

[40] Hughes L, King L, Morgan M, Ayis SA, Direkze N, Lomer M, et al. Food-related Quality of Life in Inflammatory Bowel Disease: Development and Validation of a Questionnaire. Volume 10. Journal of Crohn’s & colitis; 2015. 10.1093/ecco-jcc/jjv192 26507859

[41] Pueschel L, Hupa-Breier K, Wedemeyer H, Lenzen H, Wiestler M. Food-related Quality of Life in patients with Inflammatory Bowel Disease: Translation and Validation of the German version of FR-QoL-29. Z Gastroenterol. 2025;63(5):477–85. 40360140 10.1055/a-2542-6781 PMC12074861

[42] Satsangi J, Silverberg MS, Vermeire S, Colombel JF. The Montreal classification of inflammatory bowel disease: controversies, consensus, and implications. Gut. 2006;55(6):749–53. 16698746 10.1136/gut.2005.082909 PMC1856208

[43] Harvey RF, Bradshaw JM. A simple index of Crohn’s-disease activity. Lancet. 1980;1(8167):514. 6102236 10.1016/s0140-6736(80)92767-1

[44] Lewis JD, Chuai S, Nessel L, Lichtenstein GR, Aberra FN, Ellenberg JH. Use of the noninvasive components of the Mayo score to assess clinical response in ulcerative colitis. Inflamm Bowel Dis. 2008;14(12):1660–6. 18623174 10.1002/ibd.20520 PMC2597552

[45] Hunt SM, Alonso J, Bucquet D, Niero M, Wiklund I, McKenna S. Cross-cultural adaptation of health measures. European Group for Health Management and Quality of Life Assessment. Health Policy. 1991;19(1):33–44. 10117390 10.1016/0168-8510(91)90072-6

[46] Cruchinho P, López-Franco MD, Capelas ML, Almeida S, Bennett PM, Miranda da Silva M, et al. Translation, Cross-Cultural Adaptation, and Validation of Measurement Instruments: A Practical Guideline for Novice Researchers. J Multidiscip Healthc. 2024;17:2701–28. 38840704 10.2147/JMDH.S419714 PMC11151507

[47] Burton Murray H, Dreier MJ, Zickgraf HF, Becker KR, Breithaupt L, Eddy KT, et al. Validation of the nine item ARFID screen (NIAS) subscales for distinguishing ARFID presentations and screening for ARFID. Int J Eat Disord. 2021;54(10):1782–92. 33884646 10.1002/eat.23520 PMC8492485

[48] Lawshe CHA, Quantitative Approach. Content Validity Personnel Psychol. 1975;28(4):563–75.

[49] Bryant E, Spielman K, Le A, Marks P, Touyz S, Maguire S. Screening, assessment and diagnosis in the eating disorders: findings from a rapid review. J Eat Disord. 2022;10(1):78. 35672777 10.1186/s40337-022-00597-8 PMC9175461

[50] Walsh JM, Wheat ME, Freund K. Detection, evaluation, and treatment of eating disorders the role of the primary care physician. J Gen Intern Med. 2000;15(8):577–90. 10940151 10.1046/j.1525-1497.2000.02439.x PMC1495575

[51] Di Giorgio FM, Modica SP, Saladino M, Muscarella S, Ciminnisi S, Almasio PL et al. Food Beliefs and the Risk of Orthorexia in Patients with Inflammatory Bowel Disease. Nutrients. 2024;16(8). 10.3390/nu16081193 PMC11054879 38674883

[52] Chistol LT, Bandini LG, Must A, Phillips S, Cermak SA, Curtin C. Sensory Sensitivity and Food Selectivity in Children with Autism Spectrum Disorder. J Autism Dev Disord. 2018;48(2):583–91. 29116421 10.1007/s10803-017-3340-9 PMC6215327

[53] Cermak SA, Curtin C, Bandini LG. Food selectivity and sensory sensitivity in children with autism spectrum disorders. J Am Diet Assoc. 2010;110(2):238–46. 20102851 10.1016/j.jada.2009.10.032 PMC3601920

[54] Riccio MP, Marino M, Garotti R, Tassiello A, Maffettone V, Pezone M, et al. Food selectivity in Autism Spectrum Disorder: implications of eating, sensory and behavioural profile. Front Psychiatry. 2025;16:1587454. 40530068 10.3389/fpsyt.2025.1587454 PMC12171170

[55] Benkel I, Arnby M, Molander U. Living with a chronic disease: A quantitative study of the views of patients with a chronic disease on the change in their life situation. SAGE Open Med. 2020;8:2050312120910350. 32341782 10.1177/2050312120910350 PMC7171994

[56] de Ridder D, Geenen R, Kuijer R, van Middendorp H. Psychological adjustment to chronic disease. Lancet. 2008;372(9634):246–55. 18640461 10.1016/S0140-6736(08)61078-8

[57] Ng R, Sutradhar R, Yao Z, Wodchis WP, Rosella LC. Smoking, drinking, diet and physical activity-modifiable lifestyle risk factors and their associations with age to first chronic disease. Int J Epidemiol. 2020;49(1):113–30. 31329872 10.1093/ije/dyz078 PMC7124486

[58] Newsom JT, Huguet N, McCarthy MJ, Ramage-Morin P, Kaplan MS, Bernier J, et al. Health behavior change following chronic illness in middle and later life. J Gerontol B Psychol Sci Soc Sci. 2012;67(3):279–88. 21983040 10.1093/geronb/gbr103 PMC3325087

[59] Quick VM, Byrd-Bredbenner C, Neumark-Sztainer D. Chronic illness and disordered eating: a discussion of the literature. Adv Nutr. 2013;4(3):277–86. 23674793 10.3945/an.112.003608 PMC3650496

[60] Quick VM, McWilliams R, Byrd-Bredbenner C. Case–control study of disturbed eating behaviors and related psychographic characteristics in young adults with and without diet-related chronic health conditions. Eat Behav. 2012;13(3):207–13. 22664398 10.1016/j.eatbeh.2012.02.003

[61] Neumark-Sztainer D, Story M, Falkner NH, Beuhring T, Resnick MD. Disordered Eating Among Adolescents With Chronic Illness and Disability: The Role of Family and Other Social Factors. Arch Pediatr Adolesc Med. 1998;152(9):871–8. 9743032 10.1001/archpedi.152.9.871

[62] Yelencich E, Truong E, Widaman AM, Pignotti G, Yang L, Jeon Y, et al. Avoidant Restrictive Food Intake Disorder Prevalent Among Patients With Inflammatory Bowel Disease. Clin Gastroenterol Hepatol. 2022;20(6):1282–e91. 34389486 10.1016/j.cgh.2021.08.009

[63] Yin T, Tu W, Li Y, Yang M, Huang L, Zhang S, et al. Risk of avoidant/restrictive food intake disorder in patients with inflammatory bowel disease: predictive value of disease phenotype, disease activity and food literacy. J Eat Disord. 2023;11(1):211. 38017504 10.1186/s40337-023-00936-3 PMC10685684

